# Leveraging Smart Bed Technology to Detect COVID-19 Symptoms: Case Study

**DOI:** 10.2196/64018

**Published:** 2025-09-17

**Authors:** Gary Garcia-Molina, Dmytro Guzenko, Susan DeFranco, Mark Aloia, Rajasi Mills, Faisal Mushtaq, Virend K Somers

**Affiliations:** 1 Sleep Number Labs, 111 N Market St., Suite 500, San Jose, CA, 95113, United States, 1 6085129475; 2 Department of Psychiatry, University of Wisconsin-Madison, Madison, WI, United States; 3 Pledge Therapeutics, Leuven, Belgium; 4 Sleep Number, Minneapolis, MN, United States; 5 Department of Medicine, National Jewish Health, Denver, CO, United States; 6 Department of Cardiovascular Medicine, Mayo Clinic, Rochester, MN, United States

**Keywords:** sleep, COVID-19, symptom detection model, disease progression, illness progression

## Abstract

**Background:**

Pathophysiological responses to viral infections such as COVID-19 significantly affect sleep duration, sleep quality, and concomitant cardiorespiratory function. The widespread adoption of consumer smart bed technology presents a unique opportunity for unobtrusive, real-world, longitudinal monitoring of sleep and physiological signals, which may be valuable for infectious illness surveillance and early detection. During the COVID-19 pandemic, scalable and noninvasive methods for identifying subtle early symptoms in naturalistic settings became increasingly important. Existing digital health studies have largely relied on wearables or patient self-reports, with limited adherence and recall bias. In contrast, smart bed–derived signals enable high-frequency objective data capture with minimal user burden.

**Objective:**

The aim of this study was to leverage objective, longitudinal biometric data captured using ballistocardiography signals from a consumer smart bed platform, along with predictive modeling, to detect and monitor COVID-19 symptoms at an individual level.

**Methods:**

A retrospective cohort of 1725 US adults with sufficient longitudinal data and completed surveys reporting COVID-19 test outcomes was identified from users of a smart bed system. Smart bed ballistocardiography-derived metrics included nightly pulse rate, respiratory rate, total sleep time, sleep stages, and movement patterns. Participants served as their own controls, comparing reference (baseline) and symptomatic periods. A two-stage analytical pipeline was used: (1) a gradient-boosted decision-tree “symptom detection model” independently classified each sleep session as symptomatic or not, and (2) an “illness-symptom progression model” using a Gaussian Mixture Hidden Markov Model estimated the probability of symptomatic states across contiguous sleep sessions by leveraging the temporal relationship in the data. Statistical analyses evaluated within-subject changes, and the model’s ability to discriminate illness windows was quantified using receiver operating characteristic metrics.

**Results:**

Out of 122 participants who tested positive for COVID-19, symptoms were detected by the model in 104 cases. Across the cohort, the model captured significant deviations in sleep and cardiorespiratory metrics during symptomatic periods compared to baseline, with an area under the receiver operating characteristic curve of 0.80, indicating high discriminatory performance. Limitations included reliance on self-reported symptoms and test status, as well as the demographic makeup of the smart bed user base.

**Conclusions:**

Smart beds represent a valuable resource for passively collecting objective, longitudinal sleep and physiological data. The findings support the feasibility of using these data and machine learning models for real-time detection and tracking of COVID-19 and related illnesses. Future directions include model refinement, integration with other health signals, and applications for population-scale surveillance of emerging infectious diseases.

## Introduction

COVID-19 is responsible for a pandemic with hundreds of millions of confirmed cases and millions of deaths worldwide [1]. The majority of patients with COVID-19 (~80%) have mild influenza-like symptoms, 15% have severe symptoms, and approximately 5% develop a critical condition [2]. COVID-19 symptoms overlap with other respiratory illnesses, such as influenza, severe acute respiratory syndrome (SARS), and Middle East Respiratory Syndrome (MERS) [3,4].

“Smart” devices capable of collecting longitudinal biosignal datasets hold promise for infectious illness monitoring [5]. Consumer devices that track biometric signals over prolonged periods, such as fitness trackers and smartwatches, may be ideal tools for this task [6]. If worn consistently, these devices can establish baseline values for biometric signals and then detect deviations from baseline, such as during illness [7,8]. When used in conjunction with predictive platforms, symptoms of COVID-19 can be detected and symptom exacerbation monitored in participants using wearable smart devices [9,10]. Some studies have demonstrated that changes from baseline in certain biometric indicators measured by these devices, including resting heart rate, sleep duration, oxygen saturation, or breathing rate, might predict the onset of COVID-19 before symptoms are present [9-18].

Sleep metrics are revealing health indicators due to sleep’s important role in immunity. Studies have shown that sleep duration affects the susceptibility to, and survival from, infectious disease [19]. Certain antimicrobial peptides and cytokines act to prolong sleep, and increased sleep during infection is protective [20]. Thus, a possible immunological function of sleep is to support host defense, which has the evolutionary advantage of preparing the immunological response during a period of low metabolic need and quiescence [21].

Conversely, sleep disturbance appears to negatively impact immune function. For example, sleep deprivation induces a temporal shift in circulating levels of interleukin-6 (IL-6), such that IL-6 is undersecreted at night and oversecreted during the day, leading to excessive levels of daytime inflammation [22]. Moreover, sleep disturbance may impair the adaptive immune response, which could compromise the effectiveness of vaccines [23,24]. Although prior research suggests that perivaccination sleep influences antibody response, evidence on whether sleep disturbances impact real-world vaccine effectiveness remains limited and sometimes inconclusive. Notably, sleep metrics such as awakening frequency have shown associations with breakthrough infections beyond traditional risk factors [25].

While reduced sleep has been associated with an increased risk of respiratory infections, such as pneumonia and susceptibility to the common cold [26,27], immune activation triggered by microbial infections has also been linked to disruptions in both non–rapid eye movement and rapid eye movement sleep stages [19].

Previously, we demonstrated the ability to use sleep metrics collected from users of a smart bed platform to detect influenza-like illness (ILI) symptoms [28,29]. Using aggregated sleep data, we investigated whether seasonal trends in ILI rates reported by the US Centers for Disease Control and Prevention (CDC) could be approximated using predictions from our ILI symptom detection model described below [28]. The model predictions correlated with the reported ILI rates in the period from January 2019 to December 2020.

The ILI symptom detection model described herein comprises 2 stages: first, the binary detection-level stage that is based on the association between disturbed sleep and immune activation due to infection; and second, a temporal-dimension stage that is based on the association between disturbed sleep and immune activation due to infection. A temporal-dimension stage based on a Gaussian Mixture Hidden Markov State transition model (GMHMM) that accounts for the fact that the transition between ill and healthy states likely depends on historical data.

The aim of this study was to leverage longitudinal, retrospective, biometric data captured using ballistocardiography signals from a consumer smart bed platform [30] along with predictive modeling to detect and monitor COVID-19 symptoms at an individual level. Importantly, the data were acquired unobtrusively during sleep.

## Methods

### Smart Bed Technology

The Sleep Number smart bed technology tracks the user’s bed presence, body movements, heart rate, and breathing rate in real time to determine the duration and quality of sleep [30]. Similar to bed sensors used in health care facilities and consumer solutions located under the mattress [31,32], the smart bed uses a single pressure sensor embedded inside the mattress’s inflatable bladder to capture high-resolution full-body ballistocardiography readings, sampled at 1 kHz [30]. This type of smart bed is currently used by several hundred thousand individuals across the United States, spanning a broad age range, and operates without the need for specific sensor calibration [33].

**Figure 1.**
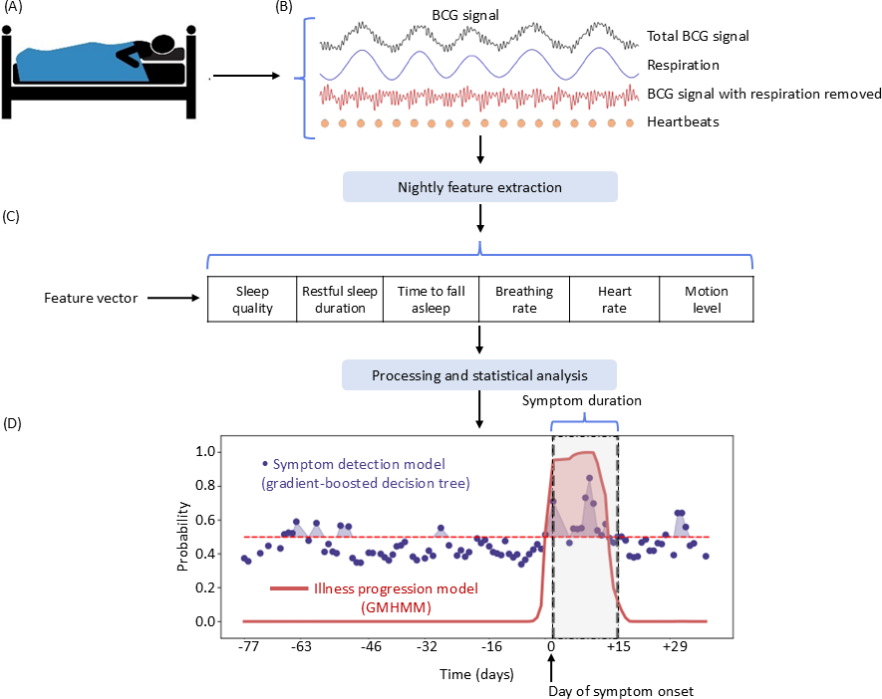
Overview of the study approach for data collection and model development. (A) The smart bed with pressure sensors collects (B) biometric signals that are used to extract (C) sleep metrics used in (D) model development. BCG: ballistocardiography; GMHMM: Gaussian Mixture Hidden Markov State transition model.

The smart bed starts collecting data as soon as the participant gets into bed and stops once they leave the bed (Figure 1A–D). Embedded software processes the data and sends the sleep metrics to the cloud [30]. Features used for this model include breathing rate (all-night mean of the instantaneous breathing rate), heart rate (all-night mean of the instantaneous heart rate), motion (mean normalized motion: difference between maximum and minimum pressure signal for 10 seconds; normalized motion is then calculated by dividing motion by the personal pressure), total sleep duration (time in bed while asleep), restful sleep duration (sleep duration minus restless time: amount of time where the motion level is higher than the motion-noise-threshold [baseline noise for an in-bed interval]), time to fall asleep (time between getting into bed and falling asleep), and sleep quality (the “Sleep IQ” score provided in the Sleep Number mobile app that correlates with sleep quality). The data are then organized into sleep sessions, defined by a continuous time spent in bed not containing any out-of-bed period longer than 2 hours.

### Survey Description and Inclusion Criteria

The survey included questions on demographics, pre-existing health conditions, general health and behaviors, and COVID-19–specific questions about exposure, testing, diagnosis, symptomatic period, and hospitalization that occurred before November 2020. The full survey is provided in the Multimedia Appendix 1. The survey collected data between August 2020 and November 2020. Of the participants who completed the survey, the respondents who provided the results of a COVID-19 test were considered for further analysis.

We included COVID-19–positive responders who satisfied the following inclusion criteria: both the start and end dates of the symptomatic period (mo/d) for their “first positive test” or “most recent test” were reported, the start date was before the end date, the available smart bed data included at least 3 sleep sessions during the symptomatic period, and the smart bed data included at least 3 sleep sessions outside the symptomatic period.

The symptomatic period was assessed based on participant responses to 2 survey items. The first item asked participants to indicate when they initially experienced symptoms and when those symptoms became most severe. The second item asked when their symptoms began to subside. Specifically, participants provided three dates: the onset of symptoms, the onset of the most severe symptoms, and the beginning of symptom resolution. COVID-19 test results obtained prior to the survey period (August 2020 to November 2020) were included in the analysis, with the testing period spanning March 2020 to November 2020.

We included COVID-19–negative responders who satisfied the following inclusion criteria: the test date (mo/d) was reported, there was no symptomatic period, the smart bed data included at least 3 sleep sessions in the COVID-19 test interval of ± 7 days, and the data included at least 3 sleep sessions outside of the test interval.

Participant cohort comparisons were made using Cohen *d* effect size. Mann-Whitney and Fisher exact tests were performed to analyze the differences between groups.

### Model Development

Figure 1A–D represents the overview of our approach, which includes the use of ballistocardiography-enabled measurement of motion, position changes, breathing, and small movements within the body, such as those generated by the ejection of blood with every heartbeat [30]. The data were organized into sleep sessions, each corresponding to 1 night of sleep. For each sleep session, we obtained the following metrics: total sleep duration, sleep quality, restful sleep duration, time to fall asleep, mean breathing rate, mean heart rate, and mean motion level (Figure 1C).

Each sleep session in either the COVID-19–positive or COVID-19–negative group included start and end dates. The end date was used to define the date to which a sleep session corresponds. Multiple sleep sessions with the same end date were merged as follows: values for breathing rate, heart rate, and motion were averaged; values for sleep duration, restful sleep duration, and time to fall asleep were summed; and the sleep quality of the combined sleep session was obtained by weighted (ie, according to sleep duration) average of the sleep quality of individual sessions. Each variable was centered by subtracting the median and dividing by the interquartile range. Such normalization was found to slightly improve the classification performance compared to the standard scaling.

We developed our detection model in 2 stages. First, a gradient-boosted, decision-tree [34] “symptom detection model” was created to classify each sleep session as symptomatic or not (Figure 1D). Second, an “ILI-symptom progression model” was built on the symptom detection model to include the temporal dimension of the symptoms and to estimate the probability of experiencing ILI symptoms (Figure 1D).

### Symptom Detection Model

The symptom detection model used self-reported data as a reference to classify each sleep session as symptomatic or not, using the range of symptomatic days provided by the survey respondents who reported a positive COVID-19 diagnosis.

Since the model detected periods of sleep metrics consistent with other ILIs, there were a few unlabeled positives in the validation data that were not specific to COVID-19. Thus, any prolonged ILI episode was an unlabeled positive in the data. As our dataset did not have negative ground truth labels (ie, ranges of dates labeled as healthy), we followed the approach in [35] and trained a propensity-weighted estimator. To create the symptom detection model, an initial estimator based on a gradient boosted decision tree [34] was used to predict the probability that a point belonging to the positive class had been assigned a positive label. Then, a second estimator was trained to classify the data into positive and negative classes using the labeling weights produced by the first estimator. These 2 estimators were iteratively trained with the Expectation Maximization algorithm until convergence [35].

The symptom detection model was used as the base model to produce the propensity score of a sleep session to be labeled as symptomatic. The hyperparameters were tuned using stratified, five-fold cross-validation to maximize a modified *F*_1_-score [36], which can be estimated using positive labels alone. The modified *F*_1_-score is defined as *F*_1_=TPR^2^/(1-NR).

TPR is the true positive rate, and NR is the negative rate of the predictions on the whole dataset. This formula prioritizes sensitivity by squaring TPR and penalizes models that over-predict negatives. Unlike the traditional *F*_1_-score, this metric does not rely on precision and recall, as the unreliability of negative ground truth labels could distort precision estimates.

### ILI-Symptom Progression Model

The “symptom detection model” was augmented with a second-level “ILI-symptom progression model” to estimate the probability of each sleep session to be symptomatic or not by leveraging the temporal dimension of the study (Figure 1). We used a GMHMM [37] because of its ability to generalize to multidimensional continuous observations and represent particular stages of several illnesses, enabling granular progression tracking.

At a given time, an individual could be in one of the two hidden states: ‘ill’ or ‘healthy.’ The estimated probability of the symptoms from the first-level model was taken as the observed emissions in the GMHMM. To detect illness, we calculated the posterior probabilities of the hidden states, which can be thought of as a smoothing filter.

The GMHMM parameters (ie, prior, transition, and emission probabilities) were also tuned to maximize the modified *F*_1_-score [36]. The simulated annealing optimizer from Python’s SciPy library [38] was used for this purpose. The input scores from the symptom detection model were calculated using a leave-one-out cross-validation approach.

### Ethical Considerations

This research was conducted in accordance with US federal guidelines (Common Rule) regulating the protection of human participants in biomedical and behavioral research. The protocol describing this research (#P2020/03001 NEIRB 17-1323071-1) was approved by the WCG New England Institutional Review Board (IRB). Participants were users of the Sleep Number smart bed. Data were collected daily. Informed consent was obtained from all participants using the Sleep IQ app (Sleep Number Corporation) on the participant’s smartphone, computer, or other electronic device of their choice. Participants gave access to nightly physiological measurements, which were electronically stored in the cloud after being anonymized. Participants completed an IRB-approved survey between August 2020 and November 2020. Participants did not receive any form of compensation for participation in this research. All collected data were anonymized before analysis, and no individual participant could be identified from the data. No images or supplementary materials contain identifiable participant information.

## Results

**Figure 2.**
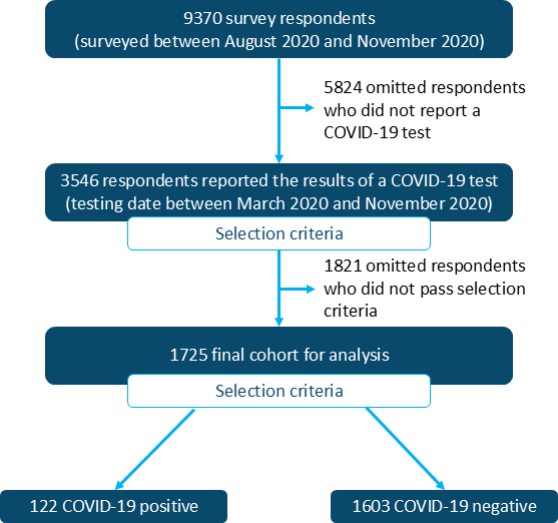
The final study cohort was composed of participants who reported a COVID-19 test result and met the inclusion criteria.

More than 9000 (N=9370) Sleep Number bed users (“participants”) responded to an IRB-approved survey from August 2020 to November 2020. Of the 9370 participants who completed the survey, 3546 (37.8%) reported results of a COVID-19 test (Figure 2).

The final cohort used in subsequent analyses was composed of 1725 participants with a known COVID-19 test outcome between March 2020 and November 2020 (Figure 2). The study cohort had a mean age of 49.5 (SD 13) years; 47.5% (820/1725) of the participants were male and had a mean BMI of 30.2 (SD 6.9) kg/m^2^. The cohort’s ethnic composition was 83.5% (1441/1725) White, 4.5% (165/3576) Black, 5.86% (101/1725) Hispanic, and 2.09% (36/1725) Asian (Table 1). In addition, 9.6% (166/1725) of the cohort smoked, 16.2% (279/1725) had asthma, 10.1% (174/1725) had diabetes, and 3.4% (58/1725) had cardiovascular disease.

**Table 1.** Participant demographics and comorbidities.

Characteristics	Survey respondents with COVID-19 test result (n=3546)	Cohort that met inclusion criteria (n=1725)	*P* value^a^^,^^b^	COVID-19–positive cohort (n=122)	COVID-19–negative cohort (n=1603)	*P* value^a^^,^^c^
Demographics						
Age (years), mean (SD)	48.0 (12.9)	49.5 (13)	<.001	45.6 (11.9)	49.8 (13.1)	<.001
Male, n (%)	1541 (43.5)	820 (47.5)	.006	49 (40.2)	771 (48.1)	.11
BMI (kg/m^2^), mean (SD)	30.5 (7.1)	30.2 (6.9)	.07	30.4 (7.4)	30.2 (6.9)	.38
Ethnicity, n (%)						
White	2850 (80.4)	1441 (83.5)	.001	100 (82)	1341 (83.7)	.61
Black	165 (4.65)	77 (4.46)	.78	3 (2.46)	74 (4.62)	.36
Hispanic	215 (6.06)	101 (5.86)	.76	13 (10.7)	88 (5.49)	.03
Asian	69 (1.95)	36 (2.09)	.66	3 (2.46)	33 (2.06)	.74
Other	247 (6.97)	70 (4.06)	<.001	3 (2.46)	67 (4.18)	.48
Comorbidities, n (%)						
Smoker	352 (9.9)	166 (9.6)	.77	8 (6.6)	158 (9.9)	.27
Asthma	591 (16.7)	279 (16.2)	.66	22 (18)	257 (16)	.53
Diabetes	343 (9.7)	174 (10.1)	.66	10 (8.2)	164 (10.2)	.54
CVD^d^	127 (3.6)	58 (3.4)	.75	3 (2.5)	55 (3.4)	.79

a Mann–Whitney *U* test (1-sided) was used to compare distributions of age and BMI. Fisher exact test (2-sided) was used to compare the rates for gender and comorbidities.

b Participants with COVID-19 (n=3546) test versus those meeting the inclusion criteria (n=1725).

c COVID-19–postive cohort (n=122) versus COVID-19–negative cohort (n=1603)

d CVD: cardiovascular disease.

Of this cohort, 7.1% (122/1725) reported a positive test result for COVID-19. There were 2 significant differences between the COVID-19–positive and COVID-19–negative groups in terms of demographic and comorbidity measures. (1) Age: the COVID-19–positive group was significantly younger than the COVID-19–negative group (mean age 45.6, SD 11.9 years vs 49.8, SD 13.1 years; Cohen *d*=0.33; *P*<.001). (2) Percent of Hispanics: the COVID-19–positive group had a significantly higher percent of Hispanic individuals than the COVID-19–negative group (13/122, 10.7% vs 88/1603, 5.49%).

There were significant differences in the demographics of the total population of survey respondents and respondents who met the inclusion criteria age (mean age 48, SD 12.9 years vs 49.5, SD 13 years; Cohen *d*=0.11; *P*<.001), gender (male 43.5 % vs female 49.2 %; *P*<.01), and percent of White individuals (83.5 % vs 80.4%; *P*=.001).

For the COVID-19–positive cohort, the symptomatic window median was 10 days and the mean was 13.7 days. In total, 1674 person-days for the symptomatic period and 18 negative sleep sessions were used to build the symptom detection and ILI-symptom progression models.

**Figure 3.**
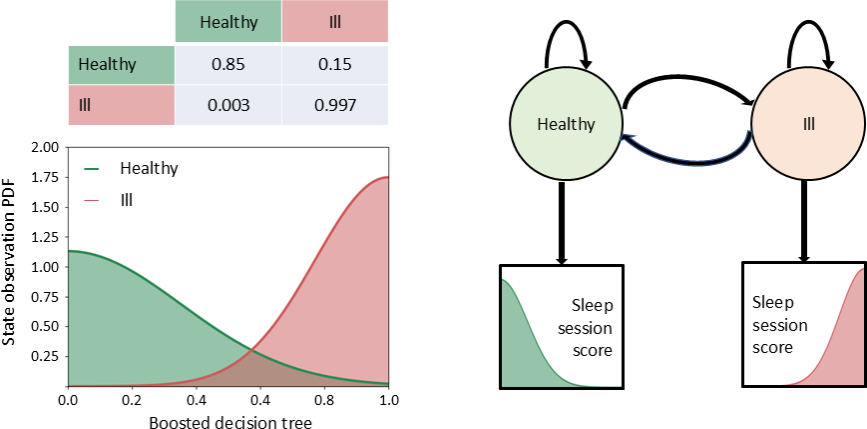
Gaussian Mixture Hidden Markov State transition model states and transition probability. PDF: probability density function.

The estimated parameters of the 2-state GMHMM model resulted in the prior probabilities and transition probabilities reported in Figure 3.

**Figure 4.**
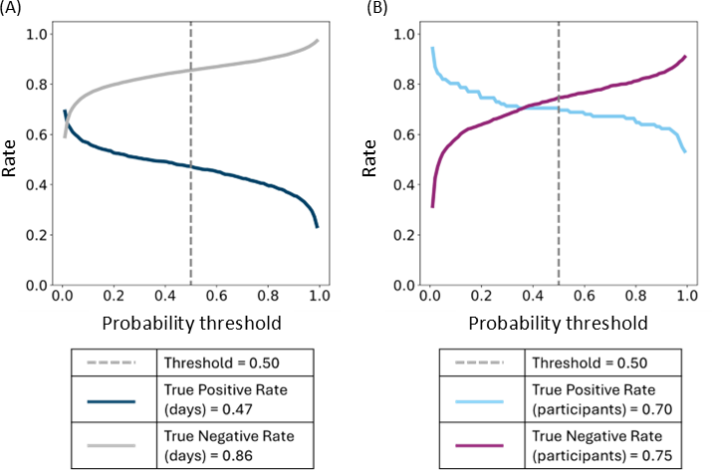
Evaluation of model performance. Symptom detection rates were calculated for (**A**) individual observed days or (**B**) participants, as a function of the probability threshold.

The combined performance of the 2-model system was evaluated as follows. First, we calculated true positive rates for the reported symptomatic days and the negative rates for the unlabeled days as a function of the probability (of being in the illness state) threshold=0.5 (Figure 4A; true positive rate: 0.47; true negative rate: 0.86; false positive rate: 0.14).

Next, we aggregated the results per participant and, as was done in the study by Krueger et al [20], recorded a successful detection if at least 1 day within the reported symptomatic interval was classified as positive (Figure 4B; true positive rate: 0.70; true negative rate: 0.75; false positive rate: 0.25). If a day or multiple days outside the participant’s self-reported symptomatic period are detected as positive by the model, the participant is categorized as negative. This approach ensures that model predictions outside the symptomatic period do not influence the classification of a participant’s overall status.

For the negative cohort, the 2-week interval around the date of the COVID-19 test administration was analyzed in the same manner as for the positive cohort. During this interval, 25% (401/1603) of the negative individuals had at least 1 day detected as positive by the model. Specifically, 401 of 1603 (25%) negative individuals were identified with at least 1 day of positive detection within the 2-week period. Conversely, 1202 of 1603 (75%) negative individuals were correctly classified as negative.

Using the 0.5 threshold on the probability, symptoms were detected in 104 of the 122 positive cases. The remaining 18 cases were categorized into 3 groups: (1) missing data before or during the symptomatic range (n=2); (2) the reported symptomatic range was abnormally short (n=2); or (3) the presence of mild symptoms was not detected by the model (n=14). Conversely, symptoms may have been detected in the negative cases due to abnormal sleep patterns not associated with a COVID-19 diagnosis, or COVID-19 tests may have been conducted too early or too late to detect a positive result (ie, a false-negative result).

**Figure 5.**
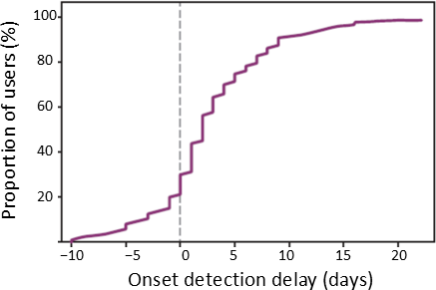
Distribution of the detected symptom onset day relative to the reported onset day.

To assess the temporal performance of our model, we calculated the detection delay (ie, the difference between the predicted first day of symptoms and the reported one). Using the 0.5 threshold, we obtained the median delay of 2 days, with 75% of the cases detected by the fifth day (Figure 5).

**Figure 6.**
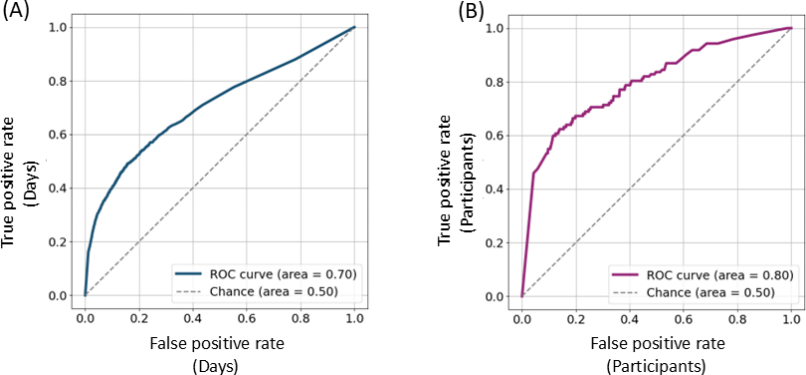
Receiving operating characteristic curve. True-positive and false-positive rates were calculated for (**A**) individual observed days or (**B**) participants as a function of the probability threshold. ROC: receiver operating characteristic.

The threshold on the probability of being in the illness state was used as a parameter to build a receiving operating characteristic (ROC) curve at a day-level (Figure 6A) and at a participant level (Figure 6B). The estimated area under the ROC curve is 0.70 and 0.80, respectively. The latter, according to the interpretation in [39], indicates that our model may have diagnostic use.

## Discussion

### Principal Findings

This study demonstrates the potential value of smart bed technology for early detection and longitudinal monitoring of influenza-like illnesses, with a focus on COVID-19, using unobtrusively collected real-world sleep and physiological data.

By leveraging a 2-stage predictive modeling approach that classifies sleep sessions and tracks symptom progression, we were able to accurately detect the onset and duration of symptoms in most individuals with a positive COVID-19 diagnosis from a large, naturalistic cohort of smart bed users. The model’s high true positive rate underscores the potential of passive, continuous biometric monitoring for supporting timely health interventions and for providing reassurance to users about evolving illness trajectories. Notably, our approach requires only routine bed use rather than the consistent wearing of a device, facilitating high adherence even during periods of illness.

### Comparison to Prior Work

Several studies have demonstrated the use of smart devices to track or predict COVID-19 symptoms [9-18]. However, these typically require consistent use of wearable devices that may prove difficult during symptomatic periods. In contrast, our method passively gathers physiological and sleep data without requiring extra effort from the user.

We have previously demonstrated the ability of a smart bed, using ballistocardiography, to detect ILIs [28]. This system has been validated against the gold standard, polysomnography, to provide reliable longitudinal sleep metrics and only requires that the user sleep in their bed consistently [30].

To our knowledge, this is the first study that considers real-world, longitudinal data collected during sleep that was unobtrusively and noninvasively acquired from a smart bed to track and detect COVID-19 symptoms. Our predictive model was built in 2 stages. First, survey data were used to classify participants into groups by self-report of negative or positive COVID-19 test results. These results were used in the symptom detection model to estimate the probability that a sleep session should be labeled “ill.” Second, the GMHMM ILI-symptom progression model was used to refine the probability estimation by incorporating the temporal dimension.

Our data analytics approach and subsequent classification model successfully detected the presence of symptoms and the duration of symptoms with a high true positive rate. As was previously demonstrated [28,29], the ability of our system to capture these endpoints is not limited to COVID-19 and can be broadly applicable to other respiratory illnesses.

### Future Directions

The sleep metrics measured with a smart bed platform are a unique source of longitudinal data, collected in a real-world and unobtrusive manner [30]. In the future, this system may serve as an asset in predicting and tracking the development of symptoms associated with a wide variety of respiratory illnesses.

A potential avenue for extending the utility of smart bed technology involves providing participants with actionable feedback about detected health anomalies, such as COVID-19–related symptoms. Recent research has demonstrated the feasibility of alert-based systems to prompt diagnostic testing in decentralized settings, combining self-reported symptoms and wearable physiological data to identify respiratory infections [40].

To better establish specificity for COVID-19 and related illnesses, future research should incorporate additional control groups representing other respiratory infections, such as influenza, SARS, and MERS.

### Limitations

There are several limitations to this study. First, the study cohort consisted entirely of Sleep Number customers, and this cohort may not be representative of the general population in terms of demographics or socioeconomic factors.

Second, a subset of the COVID-19–positive participants did not consistently sleep on their smart bed during the duration of their illness, indicating that as illness arises, smart device usage may change. Decreases in the smart bed usage were likely due to COVID-19 guidelines that suggested sleep isolation to mitigate the spread of illness among members of a household. Furthermore, as changes in sleep metrics are associated with general immune responses, the observed changes could represent several illnesses, respiratory or otherwise [10,41].

Third, several unlabeled positives representing other potential ILIs or undiagnosed COVID-19 infections were used in model validation. Hence, our “true negative rate” is biased and is more appropriately called “negative rate,” as is conventional in positive-unlabeled machine learning. Furthermore, it is possible that negative test results could have been obtained when COVID-19 testing was conducted outside of the window in which a positive result would have been detected.

Fourth, the study did not rely on objective data alone; instead, retrospective recall was required to identify COVID-19–positive symptom periods. The latter may introduce recall bias, particularly regarding the accuracy of reported illness periods. The 2-stage model used in this research refines the model predictions, which can mitigate the bias to some extent, but its influence cannot be entirely eliminated.

Fifth, the specificity of our approach for COVID-19 remains to be validated, as other illnesses —such as influenza, SARS, and MERS—exhibit similar symptomatology. To test the specificity of COVID-19 symptom detection, future research should incorporate a control group with respiratory infections exhibiting similar symptoms to COVID-19. Furthermore, while our model accounts for COVID-19–related symptoms, it does not explicitly address the influence of external factors, such as stress, anxiety, or other health conditions, which are known to affect sleep metrics (eg, sleep duration and fragmentation) [42]. These factors could introduce variability in the observed sleep patterns, potentially influencing the model’s predictions. Future studies should consider collecting and incorporating data on mental health, comorbidities, and other relevant factors to better isolate the effects of COVID-19 on sleep metrics and improve the robustness of the approach.

### Conclusions

This study demonstrates the feasibility of using smart bed–derived, longitudinal sleep and physiological data to detect and monitor self-reported symptoms of COVID-19. Our 2-stage modeling approach identified deviations in sleep duration, heart rate, and breathing rate during illness periods, correctly detecting symptoms in 104 out of 122 COVID-19–positive cases. The model achieved an area under the receiver operating characteristic curve value of 0.80 at the participant level, supporting the use of passive, in-bed monitoring for illness detection in naturalistic settings.

## Supplementary material

10.2196/64018 Multimedia Appendix 1 Survey questions and instrument used to collect self-reported COVID-19 test status, symptoms, and related health data.

## Abbreviations

CDC: US Centers for Disease Control and Prevention
GMHMM: Gaussian Mixture Hidden Markov State transition model
IL-6: interleukin-6
ILI: influenza-like illness
IRB: institutional review board
MERS: Middle East Respiratory Syndrome
ROC: receiver operating characteristic
SARS: severe acute respiratory syndrome

## References

[1] Koh HK, Geller AC, VanderWeele TJ (2021). Deaths from COVID-19. JAMA.

[2] Wu Z, McGoogan JM (2020). Characteristics of and important lessons from the coronavirus disease 2019 (COVID-19) outbreak in China: summary of a report of 72 314 cases from the Chinese Center for Disease Control and Prevention. JAMA.

[3] Larsen JR, Martin MR, Martin JD, Kuhn P, Hicks JB (2020). Modeling the onset of symptoms of COVID-19. Front Public Health.

[4] Hu T, Liu Y, Zhao M, Zhuang Q, Xu L, He Q (2020). A comparison of COVID-19, SARS and MERS. PeerJ.

[5] Seshadri DR, Davies EV, Harlow ER (2020). Wearable sensors for COVID-19: a call to action to harness our digital infrastructure for remote patient monitoring and virtual assessments. Front Digit Health.

[6] Zhu G, Li J, Meng Z (2020). Learning from large-scale wearable device data for predicting the epidemic trend of COVID-19. Discrete Dyn Nat Soc.

[7] Li X, Dunn J, Salins D (2017). Digital health: tracking physiomes and activity using wearable biosensors reveals useful health-related information. PLoS Biol.

[8] Grzesiak E, Bent B, McClain MT (2021). Assessment of the feasibility of using noninvasive wearable biometric monitoring sensors to detect influenza and the common cold before symptom onset. JAMA Netw Open.

[9] Natarajan A, Su HW, Heneghan C (2020). Assessment of physiological signs associated with COVID-19 measured using wearable devices. NPJ Digit Med.

[10] Mayer C, Tyler J, Fang Y (2022). Consumer-grade wearables identify changes in multiple physiological systems during COVID-19 disease progression. Cell Rep Med.

[11] Mishra T, Wang M, Metwally AA (2020). Pre-symptomatic detection of COVID-19 from smartwatch data. Nat Biomed Eng.

[12] Alavi A, Bogu GK, Wang M (2022). Real-time alerting system for COVID-19 and other stress events using wearable data. Nat Med.

[13] Miller DJ, Capodilupo JV, Lastella M (2020). Analyzing changes in respiratory rate to predict the risk of COVID-19 infection. PLoS ONE.

[14] Yamagami K, Nomura A, Kometani M (2021). Early detection of symptom exacerbation in patients with SARS-CoV-2 infection using the fitbit charge 3 (DEXTERITY): pilot evaluation. JMIR Form Res.

[15] Quer G, Radin JM, Gadaleta M (2021). Wearable sensor data and self-reported symptoms for COVID-19 detection. Nat Med.

[16] Hirten RP, Danieletto M, Tomalin L (2021). Use of physiological data from a wearable device to identify SARS-CoV-2 infection and symptoms and predict COVID-19 diagnosis: observational study. J Med Internet Res.

[17] D’Haese PF, Finomore V, Lesnik D (2021). Prediction of viral symptoms using wearable technology and artificial intelligence: a pilot study in healthcare workers. PLoS ONE.

[18] Liu S, Han J, Puyal EL (2022). fitbeat: COVID-19 estimation based on wristband heart rate using a contrastive convolutional auto-encoder. Pattern Recognit.

[19] Besedovsky L, Lange T, Haack M (2019). The sleep-immune crosstalk in health and disease. Physiol Rev.

[20] Krueger JM, Majde JA (1994). Microbial products and cytokines in sleep and fever regulation. Crit Rev Immunol.

[21] Irwin MR (2019). Sleep and inflammation: partners in sickness and in health. Nat Rev Immunol.

[22] Vgontzas AN, Papanicolaou DA, Bixler EO (1999). Circadian interleukin-6 secretion and quantity and depth of sleep. J Clin Endocrinol Metab.

[23] Taylor DJ, Kelly K, Kohut ML, Song KS (2017). Is insomnia a risk factor for decreased influenza vaccine response?. Behav Sleep Med.

[24] Lange T, Dimitrov S, Bollinger T, Diekelmann S, Born J (2011). Sleep after vaccination boosts immunological memory. J Immunol.

[25] Jaiswal SJ, Gadaleta M, Quer G (2024). Objectively measured peri-vaccination sleep does not predict COVID-19 breakthrough infection. Sci Rep.

[26] Patel SR, Malhotra A, Gao X, Hu FB, Neuman MI, Fawzi WW (2012). A prospective study of sleep duration and pneumonia risk in women. Sleep.

[27] Cohen S, Doyle WJ, Alper CM, Janicki-Deverts D, Turner RB (2009). Sleep habits and susceptibility to the common cold. Arch Intern Med.

[28] Guzenko D, Garcia G, Siyahjani F (2021). 651 longitudinal, unobtrusive, and ecologically valid sleep metric estimation from a smart bed to predict the pathology of COVID-19. Sleep.

[29] Guzenko D, Molina GG, Mills R, Mushtaq F (2022). Approximation of influenza-like illness rates using sleep and cardiorespiratory data from a smart bed. Sleep Med.

[30] Siyahjani F, Garcia Molina G, Barr S, Mushtaq F (2022). Performance evaluation of a smart bed technology against polysomnography. Sensors (Basel).

[31] Migliorini M, Kortelainen JM, Pärkkä J, Tenhunen M, Himanen SL, Bianchi AM (2014). Monitoring nocturnal heart rate with bed sensor. Methods Inf Med.

[32] Ranta J, Aittokoski T, Tenhunen M, Alasaukko-oja M (2019). EMFIT QS heart rate and respiration rate validation. Biomed Phys Eng Express.

[33] Garcia-Molina G, Chellamuthu V, Le B, Aloia M, Wu M, Mills R (2024). Observational study to understand the effect of timing and regularity on sleep metrics and cardiorespiratory parameters using data from a smart bed. Chronobiol Int.

[34] Chen T, Guestrin C XGBoost: a scalable tree boosting system.

[35] Bekker J, Robberechts P, Davis J, Brefeld U, Fromont E, Hotho A, Knobbe A, Maathuis M, Robardet C (2020). Machine Learning and Knowledge Discovery in Databases ECML PKDD 2019 Lecture Notes in Computer Science.

[36] Lee WS, Liu B Learning with positive and unlabeled examples using weighted logistic regression.

[37] Bilmes JA (1998). A Gentle Tutorial of the EM Algorithm and Its Application to Parameter Estimation for Gaussian Mixture and Hidden Markov Models.

[38] Virtanen P, Gommers R, Oliphant TE (2020). SciPy 1.0: fundamental algorithms for scientific computing in python. Nat Methods.

[39] Çorbacıoğlu ŞK, Aksel G (2023). Receiver operating characteristic curve analysis in diagnostic accuracy studies: a guide to interpreting the area under the curve value. Turk J Emerg Med.

[40] Quer G, Coughlin E, Villacian J (2024). Feasibility of wearable sensor signals and self-reported symptoms to prompt at-home testing for acute respiratory viruses in the USA (DETECT-AHEAD): a decentralised, randomised controlled trial. Lancet Digit Health.

[41] Prather AA, Leung CW (2016). Association of insufficient sleep with respiratory infection among adults in the United States. JAMA Intern Med.

[42] Oakes DJ, Pearce HA, Roberts C (2022). Associations between comorbid anxiety and sleep disturbance in people with bipolar disorder: findings from actigraphy and subjective sleep measures. J Affect Disord.

