# Supplementary material for: Leveraging Smart Bed Technology to Detect COVID-19 Symptoms: Case Study
Source: JMIR AI. 2025 Sep 17;4:e64018. doi: 10.2196/64018 (PMC12452045; doi:10.2196/64018)
Supplement: Multimedia Appendix 1 [file ai-v4-e64018-s001.doc]

**Survey Outline:**

1. Intro & Consent Form (Q1–Q4)
2. Testing & Diagnosis (Q5–Q11) symptoms
3. Symptoms & Hospitalization (Q12–28)
4. Exposure / Household Exposure (Q29–33)
5. General Health & Behaviors (Q34–38)
6. Pre-Existing Health Conditions (Q39–46)
7. Demographics (Q47–56)

**Q1 Welcome to the Sleep Number COVID-19 Study**
 

Sleep Number is looking to understand the relationship between sleep and critical health issues like coronavirus (COVID-19). Participating in this study will help Sleep Number to better understand the relationship between sleep and health while contributing to advancements in sleep and health research for the world. 

Depending on your responses, the survey will take approximately 15-25 minutes to complete. 


If you would like to continue and take the survey, please review and confirm your agreement with the study consent below.

**Q2 Please confirm your agreement with the consent terms.**

- I agree to the consent terms listed

**Q3 Please sign below to accept the consent terms.**

*Consent form was included*

**Q4 What is your age (in years)?**

*Write in response*

**Q5 Have you ever been tested for COVID-19?**

- No (1)
- Yes (2)

**Q6 Have you ever tested positive for COVID-19?**

- No, I tested negative (1)
- Yes, I tested positive (2)
- My results are pending (3)

**Q7 Do you think you have had COVID-19?**

- No (1)
- Yes (2)
- Maybe (3)

**Q8 How were you most recently tested for COVID-19?**

- Nasal swab (1)
- Throat swab (2)
- Saliva test (3)
- Blood sample (4)

**Q9 How were you tested when you first tested positive for COVID-19?**

- Nasal swab (1)
- Throat swab (2)
- Saliva test (3)
- Blood sample (4)

**Q10 What type of test was conducted when you were most recently tested for COVID-19?**

- Diagnostic test (shows if you have an active coronavirus infection) (1)
- Antibody test (shows if you've been infected by coronavirus in the past) (2)
- I don't know (3)

**Q11 What type of test was conducted when you first tested positive for COVID-19?**

- Diagnostic test (shows if you have an active coronavirus infection) (1)
- Antibody test (shows if you've been infected by coronavirus in the past) (2)
- I don't know (3)

**Q12 Has a medical professional ever told you that you may have COVID-19?**

- No (1)
- Yes (2)

**Q13 To the best of your ability, approximately when did a medical professional tell you that you may have COVID-19?**

Month/Day/Year: *Write in response*

**Q14 Since January 1, 2020, have you been sick for more than one day with a new illness related to COVID-19 or flu-like symptoms?**

- No (1)
- Yes (2)

**Q15 Overall, how would you rate the severity of your illness?**

- Mild: Mild symptoms effectively managed at home (1)
- Moderate: Moderate symptoms effectively managed at home (2)
- Severe: Severe symptoms requiring brief hospitalization (3)
- Very Severe: Severe symptoms requiring hospitalization and breathing support (e.g. ventilator or oxygen) (4)

**Q16 Overall, how would you rate the severity of your COVID-19 illness?**

- I experienced no symptoms (1)
- Mild: Mild symptoms effectively managed at home (2)
- Moderate: Moderate symptoms effectively managed at home (3)
- Severe: Severe symptoms requiring brief hospitalization (4)
- Very Severe: Severe symptoms requiring hospitalization and breathing support (e.g. ventilator or oxygen) (5)

**Q17 Were you admitted to the intensive care unit (ICU) when you were hospitalized?**

- Yes (1)
- No (2)
- N/A: I was not hospitalized at all (3)

**Q18 Which breathing treatment(s) did you receive? (Select all that apply)**

- Oxygen (through an oxygen mask or tube under your nose, no pressure applied) (1)
- Oxygen (through an oxygen mask, which pushes oxygen into your lungs) (2)
- A breathing machine (ventilator) with a tube down your throat (3)
- Other breathing treatment (please describe) (4)
- N/A: I did not receive any breathing treatment (5)

**Q19 How many days were you hospitalized?**

Hospitalized (in total) (1): *Write in response*

Hospitalized in intensive care unit (ICU) (2): *Write in response*

**Q20 Which of the following symptoms did you have? (Select all that apply)**

- Fever or chills (1)
- Cough (2)
- Shortness of breath (difficulty breathing) (3)
- Sleepiness / fatigue (4)
- Muscle or body aches (5)
- Headaches (6)
- New loss of taste or smell (7)
- Sore throat (8)
- Congestion or runny nose (9)
- Nausea or vomiting (10)
- Diarrhea (11)
- Other (please describe) (12) ________________________________________________

**Q21 Please rate the severity of the symptom(s) you experienced.**

|  | Extremely mild | Extremely severe |
| --- | --- | --- |

|  | 0 | 1 | 2 | 3 | 4 | 5 | 6 | 7 | 8 | 9 | 10 |
| --- | --- | --- | --- | --- | --- | --- | --- | --- | --- | --- | --- |

| Fever or chills (1) | 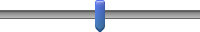 |
| --- | --- |
| Cough (2) | 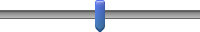 |
| Shortness of breath (difficulty breathing) (3) | 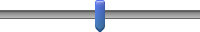 |
| Sleepiness / fatigue (4) | 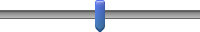 |
| Muscle or body aches (5) | 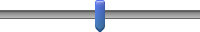 |
| Headaches (6) | 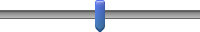 |
| New loss of taste or smell (7) | 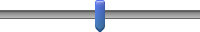 |
| Sore throat (8) | 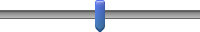 |
| Congestion or runny nose (9) | 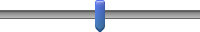 |
| Nausea or vomiting (10) | 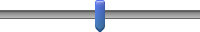 |
| Diarrhea (11) | 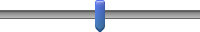 |
| Other (12) | 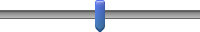 |

**Q22 What time of the day were your symptoms typically most severe?**

|  | Daytime / early afternoon   (8am-4pm) (1) | Afternoon /  evening   (4pm-12am) (2) | Nighttime / early morning (12am-8am) (3) |
| --- | --- | --- | --- |
| Fever or chills (1) |  |  |  |
| Cough (2) |  |  |  |
| Shortness of breath (difficulty breathing) (3) |  |  |  |
| Sleepiness / fatigue (4) |  |  |  |
| Muscle or body aches (5) |  |  |  |
| Headaches (6) |  |  |  |
| New loss of taste or smell (7) |  |  |  |
| Sore throat (8) |  |  |  |
| Congestion or runny nose (9) |  |  |  |
| Nausea or vomiting (10) |  |  |  |
| Diarrhea (11) |  |  |  |
| Other (12) |  |  |  |

**Q23 What is the status of your illness now?**

- I am recovered and symptom free (1)
- I am feeling better, but not completely recovered (i.e., have symptoms that remain) (2)
- I am not feeling better (3)

**Q24 Approximately, when did you first test positive for COVID-19?**

Month/Day/Year: *Write in response*

**Q25 Approximately, when were you most recently tested for COVID-19?**

Month/Day/Year: *Write in response*

**Q26 Approximately when did you first experience a symptom and when did your symptom(s) become most severe?**

First date of symptom(s): Month/Day/Year

First date of most severe symptom(s): Month/Day/Year

**Q27 Approximately when did your symptom(s) begin to subside?**

Month/Day/Year: *Write in response*

**Q28 Did you sleep in a bed other than your Sleep Number bed while you were ill?**

- Yes, for all of this time (1)
- Yes, for some of this time (2)
- No, I slept in my Sleep Number bed every night (3)

**29 In the two weeks prior to developing COVID-19 or flu-like symptoms, did you do any of the following? (Select all that apply)**

- Attended a social gathering with less than 10 people (1)
- Attended a social gathering with more than 10 people (2)
- Attended a large event (e.g. sporting event, concert, church) (3)
- Traveled outside of your state (4)
- Traveled outside of your country (5)
- Traveled on an airline (6)
- Traveled on a cruise ship (7)
- Cared for someone in nursing home or long term care facility who was known or suspected to have COVID-19 (8)
- Visited a friend, family member, coworker, or acquaintance who was known or suspected to have COVID-19 (9)
- Lived with someone who was known or suspected to have COVID-19 (10)
- Worked in a health care setting and cared for patients who were known or suspected to have
  COVID-19 (11)
- None of the above (12)

**Q30 In the two weeks prior to first testing positive for COVID-19, did you do any of the following? (Select all that apply)**

- Attended a social gathering with less than 10 people (1)
- Attended a social gathering with more than 10 people (2)
- Attended a large event (e.g. sporting event, concert, church) (3)
- Traveled outside of your state (4) Traveled outside of your country (5)
- Traveled on an airline (6)
- Traveled on a cruise ship (7)
- Cared for someone in nursing home or long term care facility who was known or suspected to have COVID-19 (8)
- Visited a friend, family member, coworker, or acquaintance who was known or suspected to have COVID-19 (9)
- Lived with someone who was known or suspected to have COVID-19 (10)
- Worked in a health care setting and cared for patients who were known or suspected to have COVID-19 (11)
- None of the above (12)

**Q31 In the two weeks prior to your most recent COVID-19 test, did you do any of the following? (Select all that apply)**

- Attended a social gathering with less than 10 people (1)
- Attended a social gathering with more than 10 people (2)
- Attended a large event (e.g. sporting event, concert, church) (3)
- Traveled outside of your state (4)
- Traveled outside of your country (5)
- Traveled on an airline (6)
- Traveled on a cruise ship (7)
- Cared for someone in nursing home or long term care facility who was known or suspected to have COVID-19 (8)
- Visited a friend, family member, coworker, or acquaintance who was known or suspected to have COVID-19 (9)
- Lived with someone who was known or suspected to have COVID-19 (10)
- Worked in a health care setting and cared for patients who were known or suspected to have COVID-19 (11)
- None of the above (12)

**Q32 Who in your household contracted COVID-19? (Select all that apply)**

- Spouse/partner (1)
- Child (2)
- Parent (3)
- Other family member (4)
- Other household member (5)

**Q33 How would you rate the severity of their COVID-19 related illness?**
 *Note: If multiple people in your household contracted COVID-19, please rate the illness severity of the person who experienced the most severe symptoms.*

|  | Not severe at all  (No symptoms experienced) (1) | Mild  (Symptoms effectively managed from home) (2) | Moderate  (Severe symptoms requiring brief hospitalization) (3) | Severe  (Severe symptoms requiring hospitalization and breathing support, like ventilator or oxygen) (4) |
| --- | --- | --- | --- | --- |
| Spouse/partner (1) |  |  |  |  |
| Child (2) |  |  |  |  |
| Parent (3) |  |  |  |  |
| Other family member (4) |  |  |  |  |
| Other household member (5) |  |  |  |  |

**Q34 What is your height?**

_______ Feet (1) *Write in response*

_______ Inches (2) *Write in response*

**Q35 What is your current weight?**

_______ Pounds (1) *Write in response*

**Q36 How often did you participate in light, moderate, and vigorous exercises (for more than 10 minutes) before the start of the COVID-19 pandemic on March, 1, 2020?**

- Light exercise: Refers to exercises that take minimal physical effort and make you breathe slightly harder than normal
- Moderate exercise: Refers to exercises that take moderate physical effort and make you breathe somewhat harder than normal
- Vigorous exercise: Refers to exercises that take hard physical effort and make you breathe much harder than normal

|  | 3 to 4 times per week (1) | 1 to 2 times per week (2) | 1 to 2 times per month (3) | Less than 1 time per month (4) | Not at all (5) |
| --- | --- | --- | --- | --- | --- |
| Light exercise (like walking) (1) |  |  |  |  |  |
| Moderate exercise (like bicycling at a regular pace, jogging) (2) |  |  |  |  |  |
| Vigorous exercise (like fast bicycling, running) (3) |  |  |  |  |  |

**Q37 How has your exercise changed, if at all, since the beginning of the COVID-19 pandemic on March 1, 2020?**

- I am exercising less (1)
- I am exercising the same (2)
- I am exercising more (3)

**Q38 Did you receive an influenza (flu) vaccine for the 2019-2020 flu season (October 1, 2019 - April 4, 2020)?**

- Yes (1)
- No (2)
- I don't know (3)

**Q39 Do you currently smoke cigarettes or use other nicotine products (eg, cigars, e-cigarettes)?**

- Yes (1)
- No (2)

**Q40 Do you have a history of any of the following conditions? (Select all that apply)**

- Asthma (1)
- Atrial Fibrillation - Intermittent (lasting less than 7 days) (2)
- Atrial Fibrillation - Persistent (lasting more than 7 days) (3)
- Cardiovascular disease (4)
- COPD (5)
- Diabetes (type 1 or type 2) (6)
- Heart attack (7)
- Heart failure (8)
- High blood pressure (9)
- Obstructive sleep apnea (10)
- Stroke (11)
- None of the above (12)

**Q41 At the time that you first developed COVID-19 or flu-related symptoms, which of the following conditions were you receiving treatment or medications for, if any? (Select all that apply)**

- Asthma (1)
- Atrial Fibrillation - Intermittent (lasting less than 7 days) (2)
- Atrial Fibrillation - Persistent (lasting more than 7 days) (3)
- Cardiovascular disease (4)
- COPD (5)
- Diabetes (type 1 or type 2) (6)
- Heart attack (7)
- Heart failure (8)
- High blood pressure (9)
- Obstructive sleep apnea (10)
- Stroke (11)
- None of the above (12)

**Q42 Do you use a pacemaker or implanted defibrillator?**

- Yes (1)
- No (2)

**Q43 Do you use a CPAP machine?**

- Yes (1)
- No (2)

**Q44 What type(s) of CPAP machine(s) do you use? (Select all that apply)**

- Nasal CPAP (air is delivered at the base of your nose) (1)
- Nasal pillow CPAP (air is delivered directly to your nostrils) (2)
- Full face CPAP (air is delivered by covering your nose and mouth) (3)

**Q45 How often do you use a CPAP machine?**

- Less than once per week (1)
- 1-2 nights per week (2)
- 3-4 nights per week (3)
- 5-6 nights per week (4)
- Every night (5)

**Q46 How many hours on average are you typically using a CPAP machine when you use it?**

- Less than 1 hour (1)
- 1-2 hours (2)
- 3-4 hours (3)
- 4-6 hours (4)
- 6-8 hours (5)
- More than 8 hours (6)

**Q47 Which side of your bed do you primarily sleep on?**

- Left side - meaning that your left arm is closest to the bed edge when laying on your back (1)
- Right side - meaning that your right arm is closest to the bed edge when laying on your back (2)
- No side in particular (3)

**Q49 What is your race/ethnicity? (Select all that apply)**

- White (1)
- Black or African American (2)
- Asian (3)
- American Indian or Alaskan Native (4)
- Hispanic or Latino (5)
- Native Hawaiian or Other Pacific Islander (6)
- Other (please specify; *Write in response*) (7)
- Prefer not to answer (8)

**Q50 What is your gender?**

- Male (1)
- Female (2)
- Non-binary (3)
- Prefer to self-describe (*Write in response*) (4)

**Q51 What is the highest level of education that you have completed?**

- Some high school or less (1)
- High school graduate (2)
- Some college but no degree (3)
- 2-year college degree (4)
- 4-year college degree (5)
- Postgraduate degree (6)

**Q52 Which of the following income categories includes your total 2019 household income before taxes?**

- Less than $15,000 (1)
- $15,000 to $24,999 (2)
- $25,000 to $34,999 (3)
- $35,000 to $49,999 (4)
- $50,000 to $74,999 (5)
- $75,000 to $99,999 (6)
- $100,000 to $124,999 (7)
- $125,000 to $149,999 (8)
- $150,000 to $199,999 (9)
- $200,000 to $249,999 (10)
- $250,000 or more (11)

**Q53 Are you currently living in the United States?**

- Yes (1)
- No (2)

**Q54 What state do you live in?**

*Dropdown menu of 50 United States*

**Q55 What city or town do you live in?**

*Write in response*

**Q56 What is your zip code?**

*Write in response*

Thank you for completing this survey and participating in Sleep Number's sleep science research! Your responses have been submitted.
